# Severe Coronavirus disease 2019 pneumonia patients showed signs of aggravated renal impairment

**DOI:** 10.1002/jcla.23535

**Published:** 2020-08-25

**Authors:** Menglu Gao, Qianying Wang, Jianhao Wei, Zhaoqin Zhu, Haicong Li

**Affiliations:** ^1^ Department of Laboratory Medicine Shanghai Public Health Clinical Center Fudan University Shanghai China

**Keywords:** COVID‐19, creatinine, ESR, hs‐CRP, procalcitonin, UA, urea, urine protein

## Abstract

**Background:**

This objective of this study was to identify a sensitive indicator of severe acute respiratory syndrome coronavirus 2 (SARS‐CoV‐2) infection.

**Methods:**

Samples were collected from 136 patients with Coronavirus disease 2019 (COVID‐19) pneumonia admitted to the Shanghai public health clinical center (116 mild, 20 severe). The concentrations of serum urea, Uric Acid (UA), Creatinine (CREA), Erythrocyte sedimentation rate (ESR), high‐sensitivity C‐reactive protein (hs‐CRP), procalcitonin (PCT), and urine protein (Pro) have been tested in this study.

**Results:**

Higher levels of urea (female 7.00 ± 3.31, male 8.87 ± 5.18) Pro (female7/7, male 12/13), hs‐CRP (female 2/7, male 5/13) ESR (female 94.43 ± 33.26, male 67.85 ± 22.77) were found in severe patients compared with the mild (urea: female 3.71 ± 1.00, male 4.42 ± 1.14; Pro: female 3/46, male 12/70; hs‐CRP: female 1/46, male 3/70; ESR: female 43.32 ± 33.24, male 21.64 ± 21.82). UA is lower in the severe group (female 146.90 ± 54.01, male 139.34 ± 66.95) than in mild group (female 251.99 ± 64.35, male 339.81 ± 71.32). CREA and PCT did not show a significant difference between mild and severe patients, but the difference among the five biological markers (urea, Pro, hs‐CRP, ESR, and UA) between mild and severe patients we tested was small (*P* < .05).

**Conclusion:**

Severe COVID‐19 patients had higher levels of urea and Pro, while their UA levels were lower, reflecting poor kidney function in severe patients. However, higher levels of hs‐CRP, ESR indicated that inflammatory responses were more active in severe patients.

## Graphical abstract

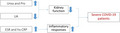

**Abbreviations**
COVID‐19: Coronavirus disease 2019
CREA: Creatinine
ESR: erythrocyte sedimentation rate
hs‐CRP: high‐sensitivity C‐reactive protein
PCT: procalcitonin
Pro: urine protein
UA: Uric Acid

## 1 INTRODUCTION

Severe acute respiratory syndrome coronavirus 2 (SARS‐CoV‐2) belongs to the β‐genus of the coronavirus family, containing an envelope of single‐stranded RNA virus that is contagious to humans.^1^ The outbreak of SARS‐CoV‐2 constitutes a public health emergency of international concern, the disease triggered by the latest coronavirus 2019 (COVID‐19) is spreading rapidly across the world.^2^, ^3^, ^4^

Recently, Charleen and his colleagues found SARS‐CoV‐2 RNA in patient stool samples and also detected in urine.^5^ Previous studies have shown that the SARS‐CoV‐2 can be transmitted via contact or fomites and also possible to spread by the fecal‐oral route.^5^ But there is no evidence of the capacity of vertical transmission.^6^ Wan et al^1^ proposed that the human‐to‐human transmission ability of 2019‐nCoV‐2 be acquired through the 2019‐nCoV‐2 RBD sequence and its receptor‐binding motif (RBM) that directly contacts ACE2. The incubation period for COVID‐19 is approximately 5‐6 days, and the serial interval range estimates from 4.4 to 7.5 days.^7^, ^8^, ^9^ Meanwhile, Zou et al^10^ pointed out the possibility that viremia might be high enough to trigger transmission for 1‐2 days before onset. Recent research has identified the evolution of SARS‐CoV‐2, leading to the outbreaks and described the potential for viral spread from animals to humans.^11^ Quarantine, social distancing, and isolation of infected populations can contain the epidemic.^12^

Fever, cough, dyspnea, and radiographic findings of pneumonia are the main clinical characteristics of patients with COVID‐19.^13^, ^14^, ^15^ Some less common symptoms have been sputum, producing headache, hemoptysis, and gastrointestinal symptoms.^13^ Evidence shows that SARS‐CoV‐2 may cause cardiac injury.^16^

Globally, there have been 12 401 262 confirmed cases of COVID‐19 by July 12, 2020, including 559 047 deaths, as reported by WHO. In particular, the major risk factors for COVID‐19 mortality are considered to be the advanced age (>60 years), male sex, and presence of comorbidities.^17^ Previous research found that acute renal impairment was uncommon in SARS but had a remarkably high mortality rate (91.7%, 33 of 36 cases).^18^ However, different results are derived based on research that showed more than 40% of patients had clinical renal disease symptoms and observed a significantly higher in‐hospital death rate in patients with renal abnormalities.^19^ Wang et al^20^ suggested that SARS‐CoV‐2 infection does not cause obvious acute renal injury or aggravate CRF in COVID‐19 patients, although it was not acceptable by others. Research conducted by Caibin Fan has suggested the damage in the renal system caused by a virus with specific renal toxicity.^21^

In order to investigate the relationship between COVID‐19 infection and renal function, multiple clinical diagnostic parameters, including CREA, UA, urea, Pro, ESR, hs‐CRP, and PCT, were compared between patients with mild and severe symptoms. We believe that the present study may help to gain a deeper understanding of the pathogenicity of SARS‐CoV‐2.

## 2 MATERIALS AND METHODS

Serum samples were collected from 136 patients with COVID‐19 pneumonia admitted to the Shanghai public health clinical center (Shanghai, China) from January 20 to January 31, 2020. Patients were divided into two groups based on their symptoms (116 mild, 20 severe). CDC has confirmed the infection of SARS‐CoV‐2. This study was approved by the Medical Ethical Committee of Shanghai public health and clinical center (NO. YJ‐2020‐S015‐01). Table 1 summarizes the demographic description of patients.

**Table 1:** Clinical characteristics of enrolled subjects

Clinical characteristic	Mild cases	Severe cases
Case	116	20
Sex (male%/female%)	70/46 (60.34/39.66)	13/7 (65.00/35.00)
Age, y [mean ± SEM]	31.20 ± 11.93	66.63 ± 13.06

Abbreviations: Male%/female%: Percentage of male/female; SEM, standard error of mean.

Serum urea, UA, and CREA concentrations were tested using ABBOTT ARCHITECT, a36000 automatic biochemical analyzer (Abbott, USA). ESR, hs‐CRP, PCT, and Pro have been detected using Vision‐B YH‐LO ESR Automatic analyzer (YH‐LO, Shenzhen, China), Lifotronic PA99 0protein analyzer (Lifotronic), Cobas 8000 Roche Automatic immune analyzer (Basel, Switzerland) and Cobas6500 Automatic analyzer (Basel, Switzerland), respectively.

All data were analyzed using SPSS 16.0 software. The independent samples *t* test was used for the data analysis of ESR, CREA, urea, UA. Samples were classified as normal and abnormal by hs‐CRP or PCT, Pro normal range, and counted the number of each group. The hs‐CRP, Pro, and PCT data were analyzed with the chi‐squared test. Graph Pad Prism version 5.0 (Graph Pad software) was used for preparing all the figures. Significant differences have been identified, as *P* < .05.

## 3 RESULTS

Due to the difference between male and female biomarkers, we compared all data in the severe and mild groups according to gender.

### 3.1 Biomarker of kidney function

#### 3.1.1 Analysis of parameters related to kidney function: Female

**Figure 1:**
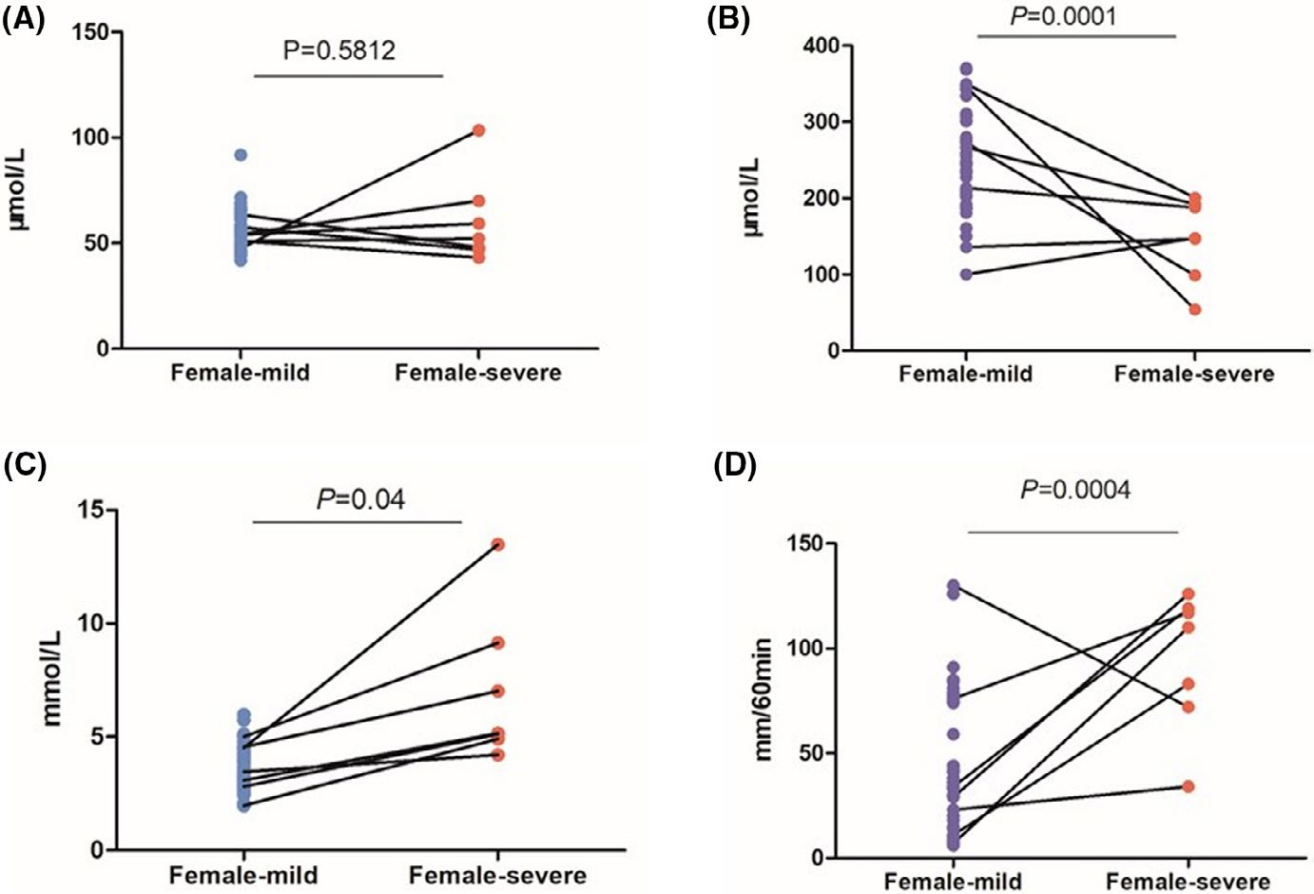
Female‐Comparison of CREA, UA, urea, ESR between severe and mild cases. A, Comparison of female CREA between severe and mild cases. B, Comparison of female UA between severe and mild cases. C, Comparison of female urea between severe and mild cases. D, Comparison of female ESR between severe and mild cases. Note: The mild, the mild group of female. The severe, the severe group of female. CREA: serum creatinine, UA: uric acid. ESR: erythrocyte sedimentation rate. *P*: *P*‐value. There was no significant difference in CREA between 2 female groups. The higher UA, ESR, and lower urea were showed in the mild cases

We obtained 53 samples from females, including 7 mild cases and 46 severe cases. Our results showed that CREA levels of the above two cases (mild 55.78 ± 9.36, severe 60.47 ± 21.00) were not significantly different (*P* = .5812, Figure 1A). The UA level was considerably lower in the severe group (146.90 ± 54.01) than in the mild group (251.99 ± 64.35; *P* = .0001, Figure 1B). The average urea level of the severe group (7.00 ± 3.31) was significantly higher than that of the mild group (3.71 ± 1.00; *P* = .04, Figure 1C). The chi‐squared test reveals the significant effects of Pro seen in severe cases (*P* < .0001, Table 2).

**Table 2:** Comparison of Pro between severe and mild cases

	Female		Male	
	Normal	Abnormal	Normal	Abnormal
The severe	0	7	1	12
The mild	43	3	58	12
Chi‐squared *P*‐value	<.0001		<.0001	

Note

Pro, urine protein. The mild, the mild group. The severe: the severe group. There were significant difference between the severe and mild groups. (Female: *P* < .0001, Male: *P* < .0001).

#### 3.1.2 Analysis of parameters related to kidney function: Male

Seventy samples were obtained from mild patients, and 13 from severe patients. The independent samples *t* test was used for evaluating quantitative variables.

**Figure 2:**
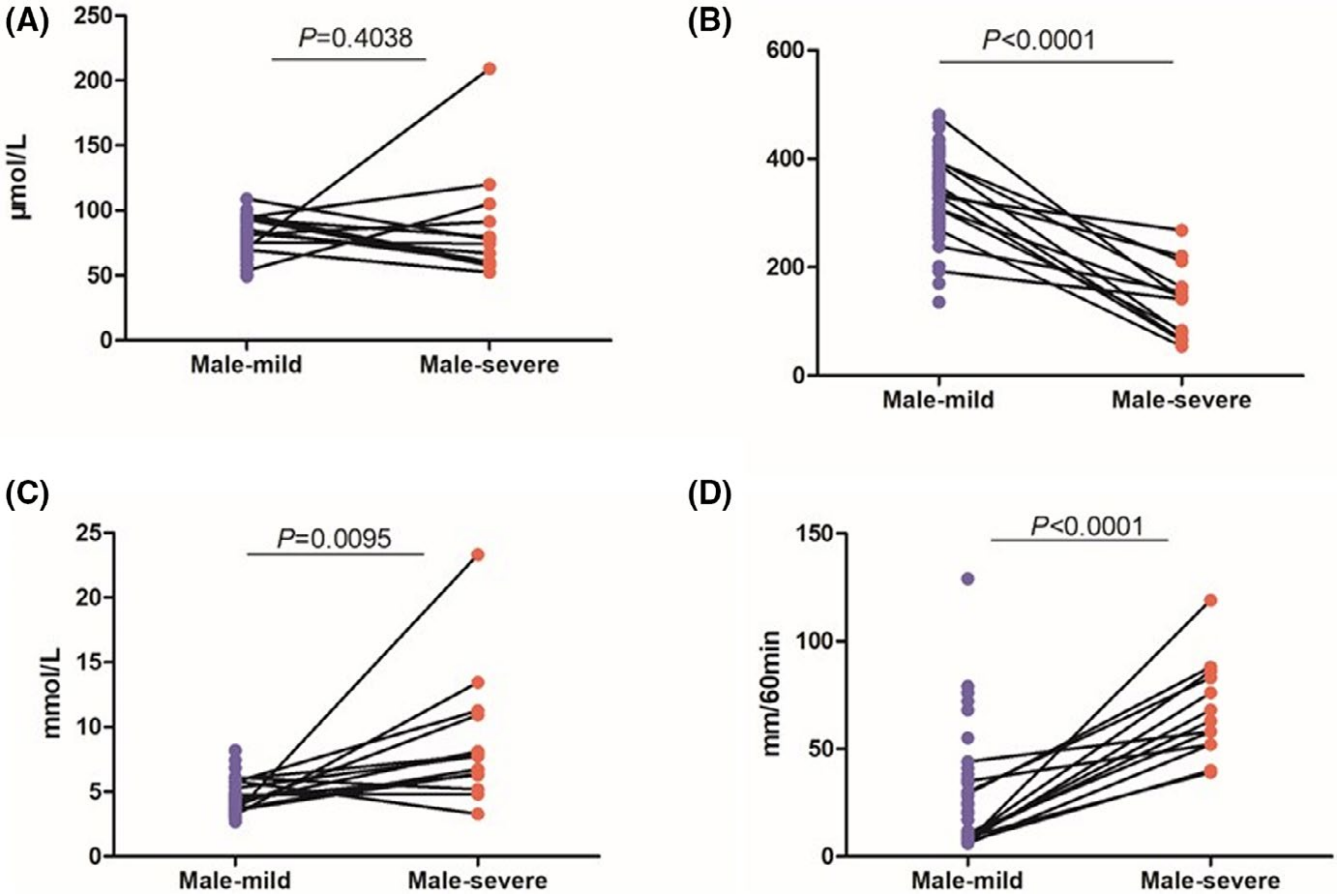
Male‐Comparison of CREA, UA, urea, ESR between severe and mild cases. A, Comparison of male CREA between severe and mild cases. B, Comparison of male UA between severe and mild cases. C, Comparison of male urea between severe and mild cases. D, Comparison of female ESR between severe and mild cases. Note: The mild, the mild group of male. The severe, the severe group of male. CREA: serum creatinine. UA, uric acid. ESR: erythrocyte sedimentation rate. *P*: *P*‐value. There was no significant difference in CREA between 2 male groups. The lower UA and higher urea, ESR were showed in the severe cases

We found no significant difference between CREA levels of two groups (mild 75.52 ± 12.74, severe 85.72 ± 42.14; *P* = .4038, Figure 2A). In contrast, the average UA level (severe 139.34 ± 66.95) in mild patients was lower (mild 339.81 ± 71.32; *P* < .0001, Figure 2B). In the severe group, the average urea level (8.87 ± 5.18) was significantly higher than the mild group (4.42 ± 1.14; *P* = .0095, Figure 2C). The chi‐squared test shows significant Pro results in severe cases (*P* < .0001, Table 2).

### 3.2 Analysis of inflammatory markers

We classified as normal and abnormal samples within the normal range of hs‐CRP or PCT. The chi‐square test analyzed the data of hs‐CRP and PCT.

#### 3.2.1 Analysis of inflammatory markers: Female

Higher levels of hs‐CRP (Table 3), ESR (94.43 ± 33.26) were found in a severe group than in mild group (ESR 43.32 ± 33.24; *P* = .0004, Figure 1D). But there is no significant difference found in PCT (*P* > .05, Table 3).

**Table 3:** Comparison of PCT and hs‐CRP between severe and mild cases

		Female		Male	
		Normal	Abnormal	Normal	Abnormal
PCT	The severe	5	2	11	2
	The mild	42	4	69	1
	Chi‐squared *P*‐value	.1704		.0625	
hs‐CRP	The severe	5	2	8	5
	The mild	45	1	67	3
	Chi‐squared *P*‐value	.0427		.0019	

Note

There was significant difference in hs‐CRP between the severe and mild groups (Female: *P* = .0335, Male: *P* = .0019). No significant difference showed in PCT between the severe and mild groups (Female: *P* = .2319, Male: *P* = .0625).

Abbreviations: hs‐CRP, hypersensitivity C ‐ reactive protein; PCT, serum procalcitonin; The mild, the mild group; The severe, the severe group.

#### 3.2.2 Analysis of inflammatory markers: Male

The hs‐CRP (Table 3) and ESR levels were significantly different between two groups (mild 21.64 ± 21.82, severe 67.85 ± 22.77; *P* < .0001, Figure 2D). The results were higher in severe cases, while there is no significant difference found in PCT (*P* > .05, Table 3).

## 4 DISCUSSION

According to a previous report, 19% (11/59) and 27% (16/59) of the COVID‐19 pneumonia patients had an elevated level of plasma creatinine and urea nitrogen, respectively.^18^ The present study showed abnormally lower levels of CREA occurring in 0.69% (5/145) of patients, as compared with the studies by Wang et al^20^ (10.8%).The CREA levels between the two groups were not significantly different. However, severe cases had considerably lower levels of UA and elevated levels of urea and Pro, compared with the mild cases, indicating that the kidney function might be compromised in severe cases. Furthermore, gastrointestinal symptoms are known to cause a rise in urea. We suspect the higher level of urea in COVID‐19 patients was caused due to SARS‐CoV‐2 related gastrointestinal dysfunction.

Previous studies of SARS‐CoV‐1 suggested that although normal plasma creatinine levels are generally observed, 6.7% of patients developed acute renal impairment occurring at a median duration of 20 days (range 5‐48 days) after viral infection.^22^

Increased levels of UA, Pro, and decreased level of urea suggested that kidney function deteriorated during the infection with COVID‐19. A recent report has shown that 100% of patients had radiographic abnormalities in their kidneys (27/27), as detected by the computerized tomography (CT) scan.^18^ Inconsistent with this result, another study found that there was an acute renal failure (ARF) in 27.06% (23/85) patients.^23^ Meanwhile, another research has shown that the most common kidney abnormalities are subclinical, and acute kidney injury (AKI) is rare (5%) in mild to moderate SARS‐CoV‐2 infection. However, AKI is most common in critically ill patients with COVID‐19.^24^ Studies have recently reported that the human kidney is a particular target for SARS‐CoV‐2 infection^23^, ^25^, ^26^ and these results might explain the reason for the abnormal kidney indicator found in the present research. The increased affinity of SARS‐CoV‐2 to ACE2 is allowing higher viral loads in several organs, particularly in the kidney, which could cause higher renal tropism of SARS‐CoV‐2 than SARS‐CoV‐1 versus.^27^

Besides, the present results showed that ESR and hs‐CRP levels also increased considerably in severe cases, which indicated stronger inflammatory reactions in severe patients than those in mild patients.

SARS‐CoV‐2 is currently under intensive investigation as a newly emerged infectious agent. Despite the limited sample size, our analysis offers essential insights into the biomarkers associated with renal function. Notably, our data highlighted that the levels of UA, Pro, and urea may serve as indicators of both the disease progression and renal function.

## CONFLICT OF INTEREST

The authors report no conflicts of interest in this work.

## ETHICAL APPROVAL

This study was approved by the Research Ethics Review Committee of the Shanghai Public Health Clinical Center (2020‐Y025‐01).
